# Bloody Stool: Is It Really Scary in Kids? Four Benign Cases

**DOI:** 10.34172/aim.2022.130

**Published:** 2022-12-01

**Authors:** Betül Öztürk, Aytaç Göktuğ, İlknur Bodur, Aysun Tekeli, Nilden Tuygun, Can Demir Karacan

**Affiliations:** ^1^Department of Pediatric Emergency Care, Dr. Sami Ulus Maternity and Child Health and Diseases Training and Research Hospital, Ankara, Turkey; ^2^Department of Pediatric Emergency Care, University of Health Sciences, Gulhane Training and Research Hospital, Ankara, Turkey; ^3^Department of Pediatric Emergency Care, Ankara City Hospital, Ankara, Turkey

**Keywords:** Bloody Stool, Cefdinir, Cephalosporin, Side effect

## Abstract

Cefdinir is a third-generation oral cephalosporin used frequently in the pediatric population. The most common side effects of cefdinir are diarrhea, nausea and dyspepsia. The side effect of turning the stool color to red and giving a bloody appearance, which is alarming for both families and physicians, is very rare. In this case report, we discussed 4 cases who referred to the emergency department with bloody stool due to the use of cefdinir. The important conclusion to be drawn from this case report is to know the rare side effects of commonly used drugs such as cefdinir. This will save time and resources and prevent unnecessary interventions on the patient.

## Introduction

 Cefdinir is a third-generation oral cephalosporin used in the pediatric population against *Staphylococcus aureus* and *Enterococcus faecalis* infections as a single or two divided daily doses with 14 mg/kg/d (maximum 600 mg) in children.^1^ It is prescribed for treatment of acute otitis media, acute pharyngitis, acute sinusitis, and uncomplicated skin lesions due to the ease of use as once-daily dosing and also for patients allergic to penicillin.^2^ The most common side effects of cefdinir are diarrhea, headache, vaginitis, and nausea. Maroon or red-colored stool is a rarely reported condition related to cefdinir use together with iron containing products.^3^

 In this case series, we report 4 patients with red stools associated with cefdinir use and iron-containing feeding (Table 1).

**Table 1:** General Characteristics of Patients with Bloody Stool

**Characteristics**	**Case #1**	**Case #2**	**Case #3**	**Case #4**
Gender	Male	Male	Male	Female
Age (months)	14	8	11	7
Complaint at emergency admission	3 times soft red stool/day	Red stool	Red stool	Red stool
Cefdinir prescribed for	Otitis Media	Otitis Media	Otitis Media	Otitis Media
Cefdinir dose (n)	7	3	1	5
Source of iron-containing product	Lactose-free formula	Formula	High-energy formula	Iron supplement
Vital findings	Stabile	Stabile	Stabile	Stabile
Examination findings	Hyperemic oropharynx and postnasal drainage	Anal fissure	Anal fissure	Hyperemic tonsils and anal fissure
Rectal examination	No blood smearing	No blood smearing	No blood smearing	No blood smearing
Gastric lavage	Normal	Normal	Normal	Normal
X-ray	Normal	Normal	Normal	Normal
Abdominal ultrasound	Normal	Normal	Normal	Normal
Fecal occult blood test	Negative	Negative	Negative	Negative
Hemoglobin at admission (mg/dL)	12.1	12.7	11.3	11
Hemoglobin at 6^th^ hours (mg/dL)	11	11.5	11.1	10.8

## Case Reports

###  Case 1

**Figure 1:**
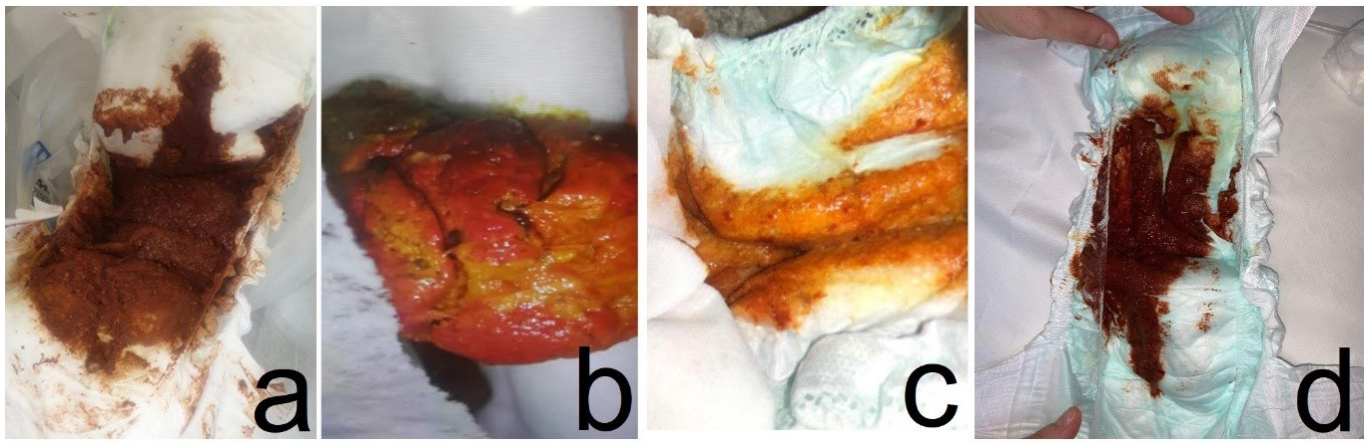
Bloody Stools of Cases

 A fourteen-month-old boy presented to the pediatric emergency department (PED) with soft red stools three times a day for the past three days (Figure 1a). Seven days ago, he was prescribed cefdinir for acute otitis media. He had been fed lactose-free formula since he was diagnosed with lactose intolerance when he was two months old. Systemic examinations were normal except for hyperemic oropharynx and postural drainage, and there was no blood smear on rectal examination. Maintenance fluid therapy was administered with an intravenous (IV) catheter, oral intake was stopped, and no blood was observed in the gastric lavage. Hemogram, coagulation parameters, C-reactive protein, electrolyte levels, and kidney and liver function tests were all in normal ranges, abdominal X-ray and ultrasonography were normal and stool occult blood test was negative. No decrease in hemoglobin was observed at the 6th hour of admission. Therefore, gastrointestinal bleeding was not considered in the patient and oral feeding was allowed. The patient was discharged. Normal colored stool was observed the day after cefdinir was discontinued.

###  Case 2

 An eight-month-old boy was brought to the PED with red colored stool (Figure 1b). The patient was taking cefdinir for acute otitis media for three days. He was allergic to ampicillin and gentamicin and was fed with breast milk and formula. He had no abnormal physical findings other than anal fissure, and there was no blood smear on his rectal examination. Gastric lavage was blood-free, he was followed up with IV fluids. There were no abnormal laboratory findings at admission and at the 6th hour, and fecal occult blood test was negative. Abdominal x-ray and ultrasonography were all normal. The patient was discharged uneventfully as he did not have recurrent bloody stools.

###  Case 3

 An eleven-month-old boy was brought to PED with bloody stool for one time (Figure 1c). Cefdinir was initiated the previous day for acute otitis media. His parents stated that he was fed a high-energy formula due to his low weight gain. He had an anal fissure, and no bloody smearing was observed on rectal examination and his laboratory findings were all normal. No fecal occult blood was detected. The patient had no hemoglobin drop; so he was discharged after the cefdinir treatment was stopped.

###  Case 4

 A seven-month-old girl was brought to PED with three times red colored stool in the same day (Figure 1d). Cefdinir was prescribed 5 days ago with the diagnosis of acute otitis media. She was fed with breast milk and iron supplements. She had hyperemic tonsils and anal fissure on examination. There was no blood smearing on rectal examination and gastric lavage was blood-free. There was no abnormality in laboratory findings, abdominal x-ray, or ultrasonography. Fecal occult blood test was negative. As there was no hemoglobin drop at 6th hour control, the patient was discharged and cefdinir treatment was stopped.

## Discussion

 Bloody stool is an emergency condition that concerns parents and poses a challenge for doctors as it may require further investigation. Here, we report 4 different cases who developed a red color change in stool after receiving cefdinir treatment with iron-containing foods.

 Although this is considered a benign condition, it can be of concern to both parents and doctors. The producer company reported the reddish stool with the use of cefdinir with iron-containing foods.^4^ Cefdinir or its metabolites combine with iron to form a precipitate that give the characteristic color change to stool. This precipitate has no further side effects and the color of the stool returns to normal after cefdinir treatment is stopped.^3^ To date, 11 cases have been reported in 9 articles related to this condition.^2,3,5^ The incidence of cefdinir-associated red stool is not clearly known.^2^ The Sanford Guide reported 1% incidence of red stool when using cefdinir and was reported to occur in approximately 10% (4 of 39) of children in a previous study.^6^

 When cefdinir is taken with iron supplements, it is thought that ferric ions form a non-absorbable complex between cefdinir or one of its metabolites, giving stool a reddish color.^3^ Iron and iron-containing products should be used two hours after cefdinir intake according to the prospectus information. Infant formula containing 2.2 mg of iron has been shown to not affect the pharmacokinetics of cefdinir and it can be mixed with these formulas.^3^ When cefdinir is given with 60 mg and 10 mg elemental iron containing formula, cefdinir absorption is reduced by 80% and 31%, respectively. However, the amount of iron taken together with cefdinir to produce red stool is not known exactly, and it is interpreted as a very low amount probably sufficient to produce red stool.^5^ Three of our four cases were fed with a formula and one case used iron supplements. The total daily iron intake of the patients in this study was 0.79 g/100 mL for the patient (Case 1) who was fed with lactose-free formula, 1.4 g/100 mL for the patient fed with high-energy formula (Case 3) and 0.5 g/100 mL for the patient who was fed with formula (Case 2). One patient used an iron supplement that contained 8 mg/mL iron a day (Case 4).

**Figure 2:**
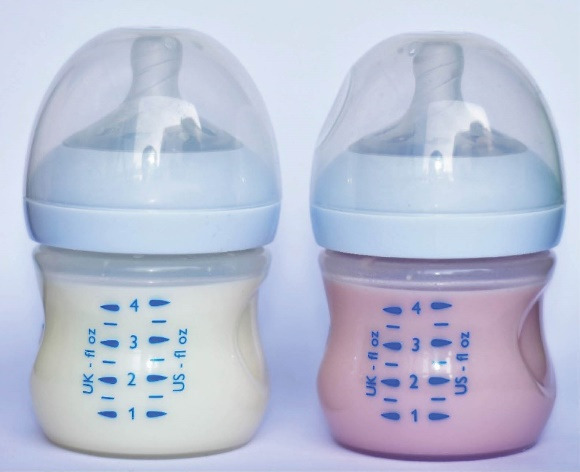
Color Change in Formula with the Addition of Cefdinir

 Patients usually use cefdinir simultaneously or mixed with the formula. In a previous case report, it was reported that PediaSure® enteral product changed color to light purple when mixed with cefdinir.^7^ Considering this report, we mixed the formulas used by the patients with cefdinir and observed that the color of the formula turned pink after a while (Figure 2). This color change is probably due to the reaction between iron and cefdinir, and families should be informed about this in advance so that they do not have unnecessary fear and anxiety.

 The onset of red stool after the use of cefdinir was reported to occur 2 to 6 days after the use of cefdinir.^2,3,5^ It was on day 3 in the first and second cases, day 5 in the third case and day 1 in the last case. In all cases, red stool did not recur after cefdinir treatment was discontinued.

 In our cases, red stool from cefdinir was estimated as highly probable by using the Naranjo scale developed to estimate the probability of adverse drug reactions.^8^ This scale is used to assess if there is a causal relationship between an identified clinical event and a drug by using a simple questionnaire to assign scores. Future studies may attempt to further investigate the incidence and onset of cefdinir-induced bloody stool.

 In conclusion, it is important to know the rare side effects of commonly used drugs such as cefdinir. There is a need for greater awareness of the side effect of cefdinir, education of patients or parents before drug administration and the appearance of red stools. The awareness of physicians about this side effect is somewhat limited. An accurate diagnosis of drug interaction could both save the time and resources that could prevent further investigation in patients with colored stools.
